# Prediction of the Effect of Methylation in the Promoter Region of ZP2 Gene on Egg Production in Jinghai Yellow Chickens

**DOI:** 10.3390/vetsci9100570

**Published:** 2022-10-16

**Authors:** Jin Zhang, Xiang-Qian Zhang, Xuan-Ze Ling, Xiu-Hua Zhao, Kai-Zhi Zhou, Jin-Yu Wang, Gen-Xi Zhang

**Affiliations:** 1 College of Animal Science and Technology, Yangzhou University, Yangzhou 225000, China; 2 Institute of Animal Husbandry, Heilongjiang Academy of Agricultural Sciences, Harbin 150001, China

**Keywords:** Jinghai yellow chickens, egg production, promoter, CpG island, methylation

## Abstract

**Simple Summary:**

Jinghai yellow Chickens have a small size, high quality, early maturity, and stress resistance characteristics. There are particular individuals in its population with high and low egg numbers at 300 days of age. As a quantitative trait, egg production is not only influenced by the environment but is also controlled by micro-effect polygenes. In this paper, we focus on the ZP2 gene, which may impact the egg production of Jinghai yellow chickens. We also analyze the role of essential methylation sites in regulating the mRNA expression of the gene and further explore the molecular regulation mechanism of the ZP2 gene.

**Abstract:**

Egg production in chickens is a quantitative trait. The aim of this study was to investigate the effect of promoter methylation of the Zona pellucida 2 (ZP2) gene on egg production. Real-time fluorescence quantification showed that the expression of the ZP2 gene in the ovaries of 300-day-old Jinghai yellow chickens in the high-laying group was significantly higher than that in the low-laying group (*p* < 0.01). A series of deletion fragments of the ZP2 gene promoter in Jinghai yellow chickens had different promoter activities in DF-1 cells, and the core region of the ZP2 gene promoter was found to be between −1552 and −1348. Four CpG islands in the promoter region of the ZP2 gene were detected by software prediction. The overall degree of methylation of the ZP2-1 amplified fragment was negatively correlated with mRNA expression to some extent (R = −0.197); the overall degree of methylation of the ZP2-2 amplified fragment was also negatively correlated with mRNA expression to some extent (R = −0.264), in which the methylation of methylcytosine (mC)-9, mC-20, and mC-21 sites was significantly negatively correlated with mRNA expression (*p* < 0.05). In addition, the mC-20 and mC-21 sites are located on the Sp1 transcription factor binding site, and it is speculated that these two sites may be the main sites for regulating transcription. In summary, the methylation sites mC-20 and mC-21 of the ZP2 gene may inhibit the binding of Sp1 and DNA, affect the transcription of the ZP2 gene, and then affect the number of eggs produced by the Jinghai yellow chickens.

## 1. Introduction

The poultry industry plays a significant role in the national economy, and the consumption of eggs by the population tends to be rigid and continues to rise. Egg production is a quantitative trait that is controlled by multiple genes, but quantitative trait loci (QTL) [1] analyses have identified regions that affect phenotypic variation in egg-related traits. Excavating candidate genes that influence egg production and uncovering their regulatory mechanisms is the pursuit of breeders.

The surface of vertebrate oocytes is wrapped in a transparent extracellular matrix as they develop, which is called the zona pellucida in mammals [2] and the protein membrane in birds, and is composed mainly of 2–4 proteins called ZONA PELLUCIDA proteins expressed by ZP (zona pellucida) genes [3]. Zona pellucida (ZP) genes are a family of genes that encode proteins that play a supportive and protective role in sperm binding and oocyte transport [4]. The number of ZP genes contained in different species varies considerably. Among mammals, humans and rabbits have four genes, ZP1–4 [5], whereas mice have three ZP genes (ZP1–ZP3) [6,7], and pigs, dogs, and cattle [8] have ZP2–ZP4. In mammals, ZP3 and ZP2 are two proteins in the acrosome response, with the former being the primary sperm receptor and the latter the secondary receptor [9]. In Zona pellucida, 42 kDa (ZPD) proteins are present in birds in addition to ZP1, ZP2, ZP3, and ZP4 [10,11,12,13,14,15,16]. Nishio et al. [17] found that chicken ZP2 is expressed in small immature oocytes and acts as a component of the blastocyst layer of mature oocytes, acting as an envelope and playing a role in the preferential binding and penetration of sperm to oocytes. Berlin et al. [18] sequenced the genes encoding gamete-recognition proteins (GRPs) in birds and revealed that four genes, including ZP2, evolved under the influence of positive selection and could therefore explain their positive selection for GRPs, such as hybridization avoidance, cryptic female choice, and postcopulatory sperm competition. Chen et al. [19] identified several differentially expressed proteins that may play a role in follicular selection in chickens, such as the ZP2 protein, by comparing proteomic analyses of SY and F6 follicles. According to the NCBI search, the chicken ZP2 gene is located on the minus strand. Until now, the association between methylation in the promoter region of the ZP2 gene and egg production in chickens has not been reported.

DNA methylation is found in bacteria, plants, and animals. In animal cells, DNA methylation often occurs at CpG dinucleotides, and the sequences of the genome that are rich in CpG dinucleotides are referred to as CpG islands. CpG islands are short dinucleotides in the genome that have high concentrations of cytosine and guanine nucleotides. Sequences with a length of more than 200 base pairs, a GC content higher than 50%, and a CpG ratio (Obs_CpG_/Exp_CpG_, O/E) greater than 0.6 are referred to as CpG islands [20]. CpG islands are randomly distributed in the genomic sequence but are usually located upstream of gene promoters and are involved in the regulation of gene expression. Approximately 60% of gene promoter regions have CpG islands, and 80% of CpGs in human and mouse genomes are methylated [21]. In specific tissues, particularly tumors, hyper-methylation of CpGs leads to increased chromosomal helicity and inhibits gene expression. There are few studies on DNA methylation in birds [22]. Because birds and mammals shared a common ancestor 300 million years ago, the chicken genome, as the first avian genome to be sequenced, will bridge a large evolutionary gap [23].

DNA methylation and histone deacetylation are the most common epigenetic phenomena. Studies on DNA methylation have focused on the promoter regions of genes to reveal the mechanisms by which their methylation status regulates their expression [24]. High DNA methylation levels can render strong promoters completely silent [25]. High levels of DNA methylation are found in its CpG island region, as confirmed by studying genes on the inactivated X chromosome. Furthermore, it has been found that if the promoter region of a gene contains a CpG island, the stronger the role that DNA methylation plays in regulating gene expression [26].

In this experiment, the expression of the ZP2 gene in ovarian tissues of high- and low-laying groups were examined by qRT-PCR using 300-day-old Jinghai yellow chickens, and a dual luciferase gene reporter system investigated the core promoter region of the ZP2 gene. This research also used BSP (Bisulfite Sequencing PCR) sequencing to quantify the methylation of the CpG island in the promoter region of the ZP2 gene in ovarian tissues and also analyzed the role of essential methylation sites in regulating the mRNA expression of the gene. In combination with the prediction of potential transcription factor binding sites in the promoter region, the molecular mechanism of the ZP2 gene regulating egg production in Jinghai yellow chickens was further explored.

## 2. Materials and Methods

### 2.1. Ethics Statement

All experimental animal protocols in this study were carried out in strict accordance with the “Jiangsu Province laboratory animal management measures”, and approved by the Animal Ethics Committee of Yangzhou University. All efforts were made to minimize animal suffering.

### 2.2. Sample Collection

The Jinghai yellow chickens were selected from the Jiangsu Jinghai Poultry Industry Group Co., Ltd. (Nantong, China). Female chickens from the same batch as the Jinghai yellow chicken resource flock with the same rearing conditions were used as candidates. The age of first egg laying (a 5% egg production rate) of this chicken breed was 130 days. At 300 days of age, the laying rate of this chicken breed was close to 70%. At 300 days of age, one high- and one low-laying individual of the same body weight and continuous egg production records were selected from each of the four semi-sibling families, with an average egg production of 134.00 ± 4.08 for the high-laying group (*n* = 4) and 50.75 ± 9.57 for the low-laying group (*n* = 4). These chickens were slaughtered. Cortex of ovarian tissues were collected, stored in liquid nitrogen, and stored at −70 °C for long-term storage. The test materials also included blood genomic DNA extracted from Jinghai yellow chickens and DF-1(chicken fibroblast cell line) cells frozen by our lab. Genomic DNA was extracted from blood using the phenol-chloroform method, and DNA concentration and purity were measured using NANODROP 1000 (Thermo Fisher, Waltham, MA, USA).

### 2.3. Primer Synthesis and Quantitative Real-Time PCR (qRT-PCR)

The qRT-PCR primers were designed according to the sequence of the chicken ZP2 gene in GenBank using Primer Premier 5.0 software (Premier Biosoft International, Palo Alto, CA, USA) (Table 1), and the primer sequence of the internal reference gene β-actin was referred to the primers designed by Zhang [27]. All primers were designed across introns to avoid contamination of genomic DNA. The primers were all synthesized by Sangon Biotech Co., Ltd. (Shanghai, China).

Total RNA was extracted from 50–100 mg of ovarian tissue using Trizol (Takara, Bao Bioengineering Co., Ltd., Dalian, China). RNA concentration and purity were measured using NANODROP 1000 (Thermo Fisher, Waltham, MA, USA). Reverse transcription cDNA synthesis was performed according to the instructions of the Reverse Transcription Kit (Takara, Bao Bioengineering Co., Ltd., Dalian, China). The reaction system contained 8 μL of PrimerScript RT Enzyme Mix, 2000 ng of total RNA, and RNase free H_2_O to 40 μL. The reaction procedure was 37 °C for 15 min, 85 °C for 5 s, and stored at 4 °C. The SYBR Green I dye kit (Takara, Bao Bioengineering Co., Ltd., Dalian, China) was used for fluorescence quantification. Three technical replicates were used for each sample. The reaction system was 20 μL: SYBR^®^Premix Ex TaqTM (2×) 10 μL, 10 μmol/L upstream and downstream primers, 0.4 μL each, ROX Reference Dye II dye (50×) 0.4 μL, template 2 μL, and 6.8 μL of sterilized distilled water to make up 20 μL. The reaction procedure for quantitative real-time PCR was: pre-denaturation at 95 °C for 30 s; denaturation at 95 °C for 5 s, annealing at 60 °C for 34 s, 40 cycles; the procedure for melting curve analysis was: 95 °C, 15 s; 60 °C, 1 min; 95 °C, 15 s; 60 °C, 15 s. We performed RT-qPCR with a five-fold ladder dilution of cDNA standards in buffer to obtain standard curves for β-actin and ZP2 genes to calculate amplification efficiency.

### 2.4. ZP2 Gene Core Promoter Region Prediction and Deletion Vector Construction

**Figure 1:**
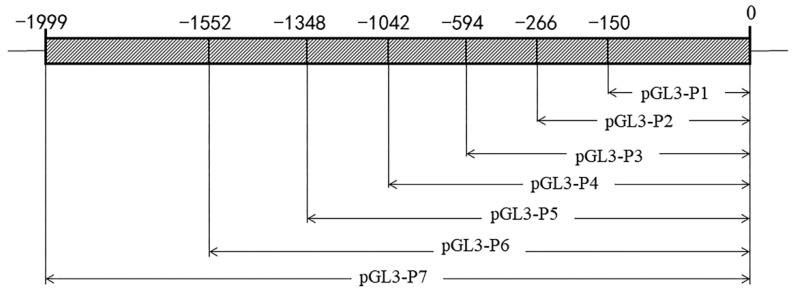
ZP2 gene deletion vector construction.

The sequence of approximately 2 kb (Supplementary Material) before the transcription start site of the chicken ZP2 gene was downloaded from the NCBI website, and core promoter prediction was performed using the Neural Network Promoter Prediction online website (http://www.fruitfly.org/seq_tools/promoter.html (accessed on 20 January 2022)). The methods and principles of prediction are based on the study of Reese et al. [28] on the promoter annotation in the Drosophila melanogaster genome. Specific amplification primers were designed for the gene promoter region using oligo7 software. The upstream and downstream primers contained Mlu I and Hind III enzyme cut sites at the 5′ end, respectively, and the downstream primer (ZPDR) was kept unchanged (Table 2). The primers were synthesized by Sangon Biotech Co., Ltd. (Shanghai, China). The amplification primers for the promoter region were verified successfully by agarose gel electrophoresis. The promoter fragments (Figure 1) were pGL3-P7 (−1999/0), pGL3-P6 (−1552/0), pGL3-P5 (−1348/0), pGL3-P4 (−1042/0), pGL3-P3 (−594/0), pGL3-P2 (−266/0), and pGL3-P1 (−150/0).

The amplification template was the genome DNA of Jinghai yellow chickens, and different segments of the chicken ZP2 gene were amplified using the designed primers. The 20 μL system was as follows: 1 μL of template DNA, 8 μL of 2 × GC-Buffer I, 2.64 μL of dNTPs (2.5 mM each), 0.8 μL each of upstream and downstream primers (10 μL/mol), and double-distilled water (ddH_2_O) were made up to 20 μL. PCR reaction procedure: 94 °C pre-denaturation for 1 min; denaturation at 94 °C for 30 s, annealing at 60 °C for 30 s, 72 °C for 1 min/kb, 31 cycles; and final extension at 72 °C for another 5 min.

### 2.5. Cell Transfection and Determination of Luciferase Activity

The ZP2 gene promoter deletion fragments pGL3-P7, pGL3-P6, pGL3-P5, pGL3-P4, pGL3-P3, pGL3-P2, pGL3-P1, negative control pGL3-basic, and the internal reference pRL-TK (Promega, USA) were co-transfected into DF-1 cells using the liposome method. When the cells were transfected for 24 h–48 h, the cells were washed twice with 1 × PBS, and then 100 μL of 1 × PLB was added to each well with a gun blast until the cells were lysed entirely and then collected into 1.5 mL centrifuge tubes for long-term storage at −70 °C. Each sample was aspirated with 10 μL of cell lysate into the enzyme standard plate. Luciferase activity was measured on a multi-mode microtiter assay system (EnSpire, Perkin Elmer, Waltham, MA, USA) according to the Dual-Luciferase ^®^Reporter Assay System (Promega, Madison, WI, USA).

### 2.6. DNA Extraction from Ovarian Tissue and Sulfite Treatment

Ovarian tissues from eight Jinghai yellow chickens selected for the experiment were used for DNA extraction using the Animal and Plant Genomic DNA Extraction Kit (AP-MN-MS-GDNA-50) (Axygen, Silicon Valley, CA, USA), and the quality of DNA extraction was detected by 3% agarose gel electrophoresis (120 V, 30 min). The samples were stored at −20 °C for backup. DNA was dissolved by sulfite reagent and methylated by the EZ DNA Methylation-GoldTM Kit (D5005) (ZYMO, Irvine, CA, USA).

### 2.7. CpG Island Analysis of Promoter Region of ZP2 Gene and BSP Primer Design

The CpG island in the promoter region of the gene was predicted using the MethPrimer online software (http://www.urogene.org/methprimer), and primers for methylation detection were designed. The related information is shown in Table 3. Finally, we used Alibaba2 to predict the potential transcription factor binding sites in the amplified fragments of ZP2-1 and ZP2-2.

### 2.8. PCR Amplification and Product Recovery

Two rounds of PCR amplification were performed using the methylated DNA as the template. The 25 μL system for the first round was: 2.5 μL of 10 × PCR Buffer, 0.75 μL of Mg2+ (50 mm), 0.5 μL of dNTPs(10 mm), 1.5 μL of Primer F(10 μm), 1.5 μL of Primer R (10 μm), 0.2 μL of Taq enzyme (5 u/μL), 2.0 μL of Template (gDNA), add ddH_2_O to make up to 25.0 μL. 50 μL system for the second round was: 5 μL of 10 × PCR Buffer, 1.5 μL of Mg2+ (50 mm), 1 μL of dNTPs (10 mm), 3 μL of Primer F (10 μm), 3 μL of Primer R (10 μm), 0.4 μL of Taq enzyme (5 u/μL), 1.0 μL of Template (first round of diluted PCR product), add ddH_2_O to make up to 50.0 μL. The PCR amplification procedure was as follows: pre-denaturation at 95 °C for 3 min; denaturation at 95 °C for 30 s; annealing at 53 °C for 30 s; extension at 72 °C for 30 s; extension at 72 °C for another 5 min; storage at 4 °C. The PCR products were detected by 1% agarose gel electrophoresis for the presence of specific target bands. Product recovery was performed according to the instructions of the SanPrep Column DNA Gel Extraction Kit (B518131) (Sangon Biotech Co., Ltd., Shanghai, China).

### 2.9. BSP Sequencing

The recovered PCR products were linked with TA vectors (Takara, Bao Bioengineering Co., Ltd., China, Dalian), and DH5α competent cells (TransGen Biotech Co., Ltd., Beijing, China) were added for monoclonal screening, and 10 clones were selected for sequencing. The sequencing kit used in this experiment was the BigDye^®^ Terminator v3.1Cycle Sequencing Kit (Thermo Fisher, Waltham, MA, USA), and the sequencing instrument was a 3730xl DNA Analyzer (Applied Biosystems, Foster City, CA, USA).

### 2.10. Statistical Analysis

For methylation level analysis for one site, the following formula was used for calculation: the number of CpG methylations in the site/the total number of CpG sites × 100%. The following formula was calculated for all sites: the total number of CpG methylations in the sites/the total number of CpG sites × 100%. A Pearson correlation analysis was performed on the degree of methylation and mRNA expression of the samples using IBM^®^ SPSS^®^ Statistics.

The qPCR results were analyzed by 2^−ΔΔCt^ method [29]. In our experiments, we defined the individual with the lowest expression in the low-laying group as the control group, and then the expression of the remaining seven individuals was compared with this control group using the 2^−ΔΔCt^ method. A *t*-test was used to analyze the differences between the high-laying and low-laying groups. Where *p* < 0.05 is marked as “*”, *p* < 0.01 is marked as “**”.

## 3. Results

### 3.1. Differential Expression Analysis of ZP2 Gene in Ovarian Tissues

**Figure 2:**
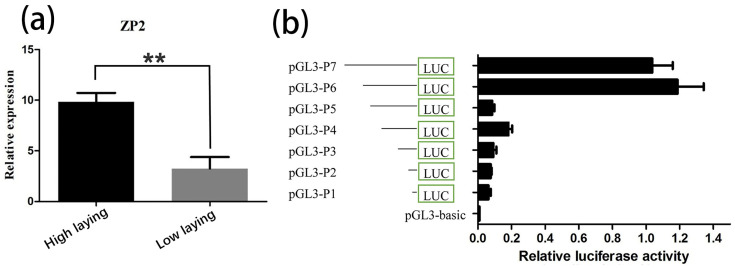
ZP2 gene expression in ovarian tissues and different promoter activity in DF-1 cells. (**a**). ZP2 gene expression in ovarian tissues of high- and low-laying Jinghai yellow chickens. “**”: Where *p* < 0.01 is marked as “**”, which means that the expression between the high-laying and low-laying groups have significant difference. (**b**). The activity of the different promoters of the ZP2 gene in DF-1 cells.

The amplification efficiency from standard curves for ZP2 gene and β-actin was E = 102.82% and E = 107.37%, which showed that the expression of the ZP2 gene can be analyzed using 2^−ΔΔCt^ method. In this experiment, the mRNA expression of the ZP2 gene in the ovarian tissues of 300-day-old Jinghai yellow chickens was compared between the high- and low-laying groups using qRT-PCR, and the analysis results are shown in Figure 2a. As can be seen from Figure 2a, the expression of the ZP2 gene was significantly higher in the high-laying group than in the low-laying group.

### 3.2. Prediction of the Core Promoter Region of ZP2 Gene and Assay of ZP2 Activity in DF-1 Cells Transfected with Deletion Vector

The promoter predicted two potential core regions using Neural Network Promoter Prediction online software (Table 4). DF-1 cells were co-transfected with the seven recombinant plasmids and pRL-TK vectors. The pGL3-basic vector plasmid was used as a negative control to detect the respective promoter activities (Figure 2b). The results showed that the ZP2 promoter fragment of Jinghai yellow chickens had different promoter activities in DF-1 cells. The activity of the recombinant plasmid pGL3-P6 was the highest. The activity of pGL3-P5 was significantly decreased, and the pGL3-P1~pGL3-P4 vectors had lower activity in the DF-1 cells.

### 3.3. ZP2 Gene CpG Island Prediction and PCR Amplification

**Figure 3:**
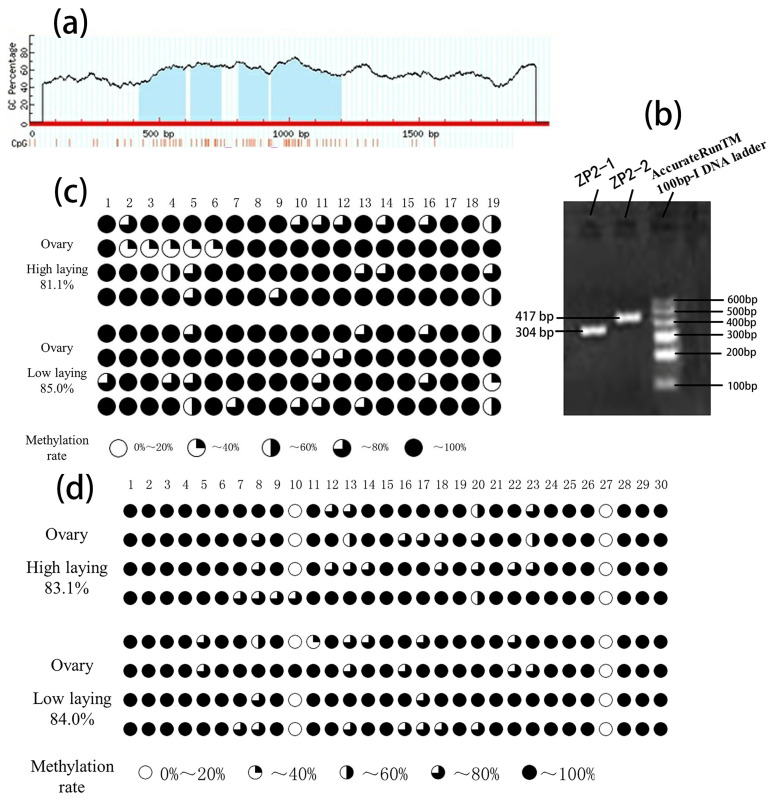
Prediction of CpG islands, examination of amplification products, and detection of methylation levels of different fragments in ovarian tissue. (**a**). Prediction of CpG islands. The four light blue regions are the predicted CpG islands in the promoter region of the ZP2 gene. The position of the horizontal coordinate “0” is the −2000 position of the sequence; similarly, the position of the coordinate “500 bp” refers to the −1500 position of the sequence, the position of the coordinate “1000 bp” refers to the −1000 position of the sequence, and the position of the coordinate “1500 bp” refers to the −500 position of the sequence. (**b**). Agarose gel electrophoresis of amplification products of the ZP2 gene using methylation primers. (**c**)**.** Methylation level of ZP2-1 fragment in the ovary. (**d**). Methylation level of ZP2-2 fragment in the ovarian. Blank circles represent 0~20% degree of methylation, a quarter of black circles represent 20~40% degree of methylation, half of the black circles represent 40~60% degree of methylation, three-quarters of black circles represent 60~80% degree of methylation, all black circles represent 80~100% degree of methylation.

MethPrimer analyzed CpG islands in the promoter region of the ZP2 gene. As shown in Figure 3a, the promoter region of the ZP2 gene contains four CpG islands, but the distance between the CpG islands is short. Combined with the results of the previous experiments, it was found that the core promoter region of the ZP2 gene (−1552~−1348) was located within the first two CpG island regions.

A pair of BSP primers were designed for the first two CpG islands and the last two CpG islands, respectively, named ZP2-1 (−1588~−1285) and ZP2-2 (−1220~−804). The CpG island of the ZP2 gene was amplified by conventional PCR using BSP primers. The amplified fragments were consistent with the expected size by 1% agarose gel electrophoresis, and the specificity was reasonable. The following sequencing can be carried out (Figure 3b).

### 3.4. BSP Sequencing Results of CpG Island Methylation of the ZP2 Gene

After BSP sequencing, the overall methylation degree and the number of methylation sites of the amplified fragment in the high- and low-laying groups were shown in Table 5. The overall methylation degree of the ZP2-1 fragment was 81.1% and 85.0% in the high- and low-laying groups, respectively, and some of the methylation sites were more discrete (Figure 3c). The overall methylation degree of the ZP2-2 fragment was 83.1% and 84.0% in the high- and low-laying groups, respectively, with mC-10 being more discrete, and the other sites were relatively conserved (Figure 3d).

### 3.5. Pearson Correlation Analysis between Methylation Level and mRNA Expression

**Figure 4:**
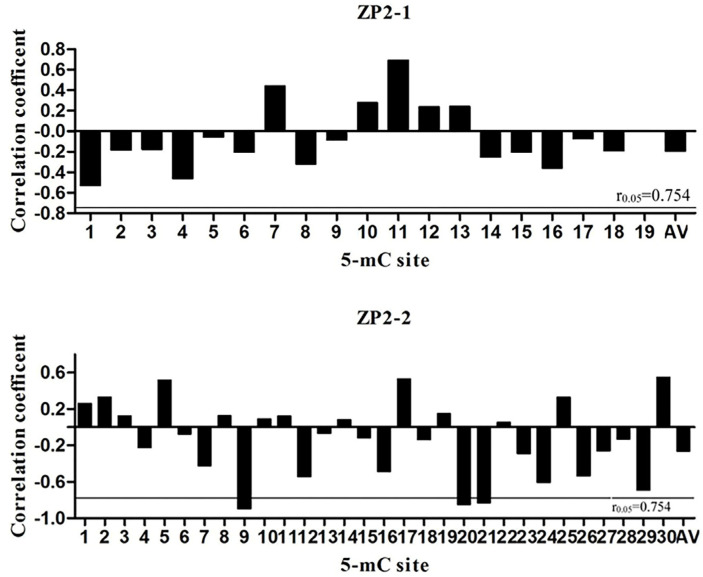
Correlation analysis between ZP2-1, ZP2-2 amplified fragment methylation, and mRNA expression level. AV: The average level of methylation. The 5-mC site: the 5-methylcytosine site formed after DNA methylation is an important epigenetic modification.

The results of Pearson analysis between methylation level and mRNA expression are shown in Figure 4. The overall methylation levels of both ZP2-1 (R = −0.197, *p* = 0.672) and ZP2-2 (R = −0.264, *p* = 0.567) fragments were negatively correlated with mRNA expression. In the ZP2-1 fragment, no single methylation site reached a significant correlation level. In contrast, a significant negative correlation between the mRNA expression and methylation level at mC-9, mC-20, and mC-21 sites for ZP2-2 was detected (*p* < 0.05).

### 3.6. Prediction of the Potential Transcription Factor Binding Sites of the ZP2 Gene

**Figure 5:**
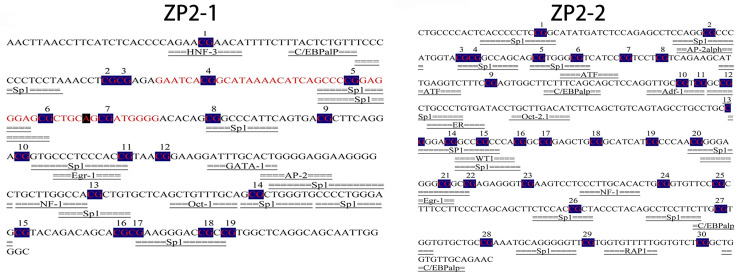
Transcription factor binding site analysis of ZP2-1 and ZP2-2 amplification fragment. The numbers in the figure are 5-mC sites: the 5-methylcytosine site formed after DNA methylation.

The potential transcription factor binding site prediction analysis of ZP2-1 (−1588~−1285) and ZP2-2 (−1220~−804) amplicons was performed using Alibaba2. The predicted results of the ZP2-1 amplicon are shown in Figure 5. Seventeen potential transcription factor binding sites, including ten Sp1 binding sites, were identified. The predicted results of the ZP2-2 amplicon are shown in Figure 5. Twenty-four potential transcription factor binding sites, including eleven Sp1 binding sites, were identified. Significantly negatively correlated with mRNA expression, mC-9 was not located within a transcription factor binding site, whereas mC-20 and mC-21 were located at the Sp1 transcription factor binding site.

## 4. Discussion

It has been shown that the ZP gene is expressed mainly in the ovary and liver, with different expression patterns in different species [30]. Xie [31] found that ZP2 mRNA was not expressed explicitly in oocytes but also in the granulosa cell layer of follicles and was most abundantly expressed in the granulosa cell layer of the luminal follicles. Nishio et al. [17] showed that the ZP2 gene was expressed mainly in the granulosa cell layer of immature white follicles. However, there was also a small amount of expression in oocytes, as illustrated by a study on Japanese quail [12,14]. In this experiment, we analyzed the expression of the ZP2 gene in the ovarian tissues of high- and low-laying groups of Jinhai yellow chickens. We found that the expression of the ZP2 gene in the high laying group was significantly higher than that in the low laying group (*p* < 0.01), which indicated that the ZP2 gene might have some influence on the egg production of Jinghai yellow chickens.

The luciferase reporter system is the most commonly used technology for detecting promoter activity. This study found that the activity of pGL3-P6 was significantly higher than that of pGL3-P5 (*p* < 0.01), indicating that the core region of the ZP2 gene promoter was located between −1552 and −1348, and the core region 1 (−1516~−1466) predicted by Neural Network Promoter Prediction was located in it. In addition, the activity of pGL3-P6 was slightly higher than that of pGL3-P7, indicating that there may be elements inhibiting promoter activity between pGL3-P6 and pGL3-P7. At the same time, there may be some positive regulatory elements between pGL3-P4 and pGL3-P3. The determination of regulatory elements needs further research.

Two mechanisms have been identified for inhibiting gene expression by promoter methylation. One is binding MBPs (methyl-binding proteins) to methylated DNA sequences, which prevents transcription factors from binding specifically to the sequence and inhibits gene expression [32,33]. The second is that the conformation of the gene is altered by methylation, which also affects the binding of transcription factors and results in the silence of the gene [34,35]. Each CpG island located in the promoter region may have many methylation sites, with certain specific CpG sites that play a crucial role in the function of CpG islands and are critical sites for the regulation of gene function [36,37,38].

In this study, BSP sequencing of the CpG island of the ZP2 gene showed that 19 and 30 CG sites were methylated in the ZP2-1 fragment and the ZP2-2 fragment, respectively. In the high- and low-laying groups of Jinghai yellow chickens, the overall methylation levels in ovarian tissue have certain differences, and mRNA expression has a negative correlation with the methylation, indicating that the methylation of the promoter region in the ZP2 gene has an inhibitory effect on gene expression. However, the ZP2-1 amplified fragment contained the core region of the gene promoter and none of its single methylation sites correlated with mRNA expression at a significant level. The ZP2-2 amplification fragment was not located in the core region. At the same time, there was a significant negative correlation between the methylation of the mC-9, mC-20, and mC-21 sites and mRNA expression (*p* < 0.05), with the mC-20 and mC-21 sites located at the Sp1 transcription factor binding site. Studies have shown that Sp1 binding to the target sequence can play a role in the initiation of transcription [39,40], and there are a large number of SP1 binding sites in the promoter [34,41] or enhancer [42,43] of the gene. In addition, mC-20 and mC-21 in the ZP2-2 fragment are located not only at the SP1 binding site but also at the Egr-1 binding site. Early growth response-1 (Egr-1) is a Cys2-His2-type zinc-finger transcription factor. A broad range of extracellular stimuli is capable of activating Egr-1, thus mediating growth, proliferation, differentiation, or apoptosis. Studies have shown that the 5’-flanking region of the Egr-1 gene contains genetic elements that are essential in connecting stimulation of the cells with enhanced transcription of the Egr-1 gene and, subsequently, transcription of Egr-1-responsive genes [44]. Five serum response elements (SRE) have been identified in the promoter region of Egr-1, the binding region of serum response factor (SRF) [45]. Our research suggests that the mC-20 and mC-21 sites of the ZP2 gene may be critical sites for regulating gene transcription, while other methylation sites may be auxiliary sites.

The study of promoters and core promoters is currently receiving increasing attention. As transcriptomics has advanced, the study of known gene expression patterns and new genes has developed considerably, and the study of the regulatory mechanisms of gene expression has become significant, especially concerning promoter region methylation and transcription factors. For example, many studies on promoters’ relevance to cancer. Ling et al. [46] showed that DNA methylation is more likely to induce cellular malignancy than promoter mutations. Studies have found promoter methylation of TRb1, which is associated with various human tumors. In addition, studies on transcription factors in the promoter region have been ongoing. Ling et al. [47] showed that adiponectin is a vital regulator of fat metabolism in the organism. CREB mainly regulates adiponectin gene transcriptional activity, and its binding site is a deletion in this gene in humans and mice. Jessica et al. review the literature relating to DNA methylation in an array of taxa and discuss these data from an ecotoxicological perspective and effects of environmental contaminants on DNA methylation in animals were also analyzed [48]. In this study, by studying the methylation of the promoter region of the ZP2 gene, we found that the degree of methylation of the ZP2 gene promoter region binding site was significantly and negatively correlated with the expression of the ZP2 gene and was located at the SP1 transcription factor binding site. Our experiments, therefore, hypothesize that methylation of part of the ZP2 gene promoter region, which is associated with egg production, affects the binding of transcription factors. In future experiments, we can continue to investigate the recognition of specific DNA sequences by transcription factors and explore the crucial effects of transcription factors on reproductive production.

## 5. Conclusions

In this study, the expression of the ZP2 gene in ovaries was found to be significantly higher in the high-laying group than in the low-laying group (*p* < 0.01). The core region of the gene was predicted to be located from −1552 to −1348 by constructing deletion reporter vectors for the promoter fragment of the ZP2 gene in the Jinghai yellow chicken. The methylation of the mC-20 and mC-21 sites in the promoter region of the ZP2 gene was significantly negatively correlated with mRNA expression (*p* < 0.05). Both the mC-20 and mC-21 sites were located at the Sp1 transcription factor binding site, which may inhibit the binding of Sp1 and DNA, affect the transcription of the ZP2 gene, and thus affect the egg production of Jinghai yellow chickens.

## Figures and Tables

**Table 1:** qRT-PCR primers and sequences.

Gene	Accession Number	Primer Sequence (5′-3′)	Product Size (bp)
ZP2	NM_001039098.1	F: TGATGTCTTGTGAGCACGCTGTAG R: GTGCCATTCAGCAGCAGGAG	121
β-actin	X00182	F: CAGCCATCTTTCTTGGGTAT R: CTGTGATCTCCTTCTGCATCC	169

**Table 2:** Primer sequences of the promoter fragment.

Fragment Name	Primer Sequence (5′-3′)	Product Size (bp)
pGL3-P7	CAGCTGTGGCTTCTCTGAGCG	1999
pGL3-P6	CTTTACTCTGTTTCCCCCCTCC	1552
pGL3-P5	GTGCCCCTGGGAGCGTACAGA	1348
pGL3-P4	TTCAGCTGTCAGTAGCCTGCC	1042
pGL3-P3	TCCCCTGTGAGGGAAGGCTTG	594
pGL3-P2	GCCTACAGGAAAGCTGGGGAG	266
pGL3-P1	GCAGTGAGACACTGGAACAGG	150
ZPDR	TTAAGACCACCTCGTTCCACCC	

**Table 3:** Primers of the methylation detection region.

Gene	Primer Sequence (5′-3′)	Product Size (bp)
ZP2-1	F: AAGTTATTTAGATTGAAAATATAAATTAGGGGAT R: CATTCATTTCCACCACAACAACAAC	304
ZP2-2	F: GGAGTTATAGGTTTTAAGAGAGGGAGG R: CCACATTACTCCATCTATACCCAAAAAC	417

**Table 4:** Prediction of the core region of ZP2 gene promoter in Jinghai Yellow Chicken.

Core Region	Start (bp)	End (bp)	Primer Sequence (5′-3′)	Score (0–1)
1	−1516	−1466	GAATCACGGCATAAAACATCAGCCCCGGAGGGAGCGCTGCAGCGATGGGG	0.96
2	−249	−198	GGAGGGACTTTTTATAGGGGCAGGTAGTGACCAGATGGCTTTAAACTGGA	0.95

**Table 5:** The methylation level of amplified fragments.

Fragment	Overall Methylation Level (%)		Number of Methylated Sites
	High Laying Group	Low Laying Group	
ZP2-1	81.1	85.0	19
ZP2-2	83.1	84.0	30

## Data Availability

Not applicable.

## Supplementary Materials

The following supporting information can be downloaded at: https://www.mdpi.com/article/10.3390/vetsci9100570/s1, Figure S1: Sequence before the transcription start site of the chicken ZP2 gene.
