# Supplementary material for: Prediction of the Effect of Methylation in the Promoter Region of ZP2 Gene on Egg Production in Jinghai Yellow Chickens
Source: Vet Sci. 2022 Oct 16;9(10):570. doi: 10.3390/vetsci9100570 (PMC9609111; doi:10.3390/vetsci9100570)
Supplement: Supplementary file 1 [file vetsci-09-00570-s001.zip › Figure S1.pdf]

>chr14:14979735-14980550
TCAGCTGTGGCTTCTCTGAGCGCACGTGGAAGTTTTAGTTGTTATCAAGGCAGAAGGGGTATTTTGAACTCACA
TCTTGAGGTTGCTCAGTTATTTGTTCCCTGGGCGGTCATTCCCTCCCAGGTGCTCTATGGACTCTCAGTACCTCC
TTGCATCCGCCTTCATCACACAAGTTAATTTTTGCTTTCCCTGGAGGTGCAGCCACAGCAGTAAAGTCCAAAGG
CCCTTTTTATTGTGCAGCCACAGCCACGCCTGAGCTCAGCCGTACAGTGGATCTGTGGCAATCCCATCTGGGTG
TGATATTTTCAAAGCTATTTCTAGGCAAGGAGAACTAAATGCGTCCGAGTAGTTCATCAAATATTCCCCCCGATC
TCCTTTAAGCCATTCAGATCGAAAACACAAACCAGGGGACAACTTAACCTTCATCTCACCCCAGAACGAACAT
TTTCTTTACTCTGTTTCCCCCCTCCTAAACCTCGCGAGAGAATCACGGCATAAAACATCAGCCCCGGAGGGAGC
GCTGCAGCGATGGGGACACAGTGGCCCATTCAGTGACGCTTCAGGACGGTGCCCTCCCACCGTAACGGAAGG
ATTTGCACTGGGGAGGAAGGGGCTGCTTGGCCACGCTGTGCTCAGCTGTTTGCAGCGCTGGGTGCCCCTGGGA
GCGTACAGACAGCACGCGAAGGGACCGCCGTGGCTCAGGCAGCAATTGGGGCGTCGCTGCTGCGGTGGAAAT
GAACGCTAATTGGGGTCGGAGCCACAGGCCCCAAGAGAGGGAGGCTGCCCCACTCACCCCCTCCGGCATATG
ATCTCCAGAGCCTCCAGGCGCCCATGGTACGCGGCCAGCAGCGTGGGCGTCATCCCGTCCTCGTCAGAAGCAT
TGAGGTCTTTCCGAGTGGCTTCTTTCAGCAGCTCCAGGTTGCCATCGGCCGCTGCCCTGTGATACCTGCTTGAC
ATCTTCAGCTGTCAGTAGCCTGCCTGCCGGGACGGCCCGCCCACGGCCGGAGCTGCGGCATCATCGCCCAACG
GGGAGGGCGGCCGAGAGGGTCGAAGTCCTCCCTTGCACACTGCGGTGTTCCCGCTTTCCTTCCCTAGCAGCTT
CTCCACTGCTACCCTACAGCCTCCTTCTTGCGTGGTGTGCTGCCGAAATGCAGGGGGTTCGTGGTGTTTTTGGT
GTCTCGGCTGGTGTTGCAGAACGTTTTTGGGTACAGATGGAGCAATGCGGCTGATTTCCTCCAGGCAGCACAG
GAGCTCCAGCAGCAGCAGCGCAGTGCTCCTGACCCAGTGGGCTGCACACGCTGCTGGTGCCCTATGGGATGGT
CCATCCGGCCTCATCCTCACGGGCACAGTTCTCAGTTTAGACATTATTTCCAGTGATGACAGTGGAGCAATGTC
CAGTGGAGCCTTCCCCTGTGAGGGAAGGCTTGTGGAACTAGAGCACAGGGTTGGGTGCTAGAAAAGACTTCT
CCAAAAGAGCGGTGATGCATTGGAACGGGCTGCCTGGGGGGAGAGGAGTCACCATCTCTGGAGGGATCCAAG
ATCCATAGAGATGATGTGGGCAGCGGGCAATATTGGTGGTAAGGGGATAATTAGACTGGATGGTCTTGCAGGCC
ATGAAGATCAGAGGATGGAGCACCTCCATGATGAGCACAGGCTGAGAGAGCTGGGGCTGTTCAGCCTGGAGA
AGAGAAGGCTCCAGAGAGGCCTTATAGTAATCTTCCAGGACCTGAAGGGGCCTACAGGAAAGCTGGGGAGGG
ACTTTTTATAGGGGCAGGTAGTGACCAGATGGCTTTAAACTGGAAGAGGGTAGATTTAGACTAGCTATTAGGAA
GAAATTCTTTATTGTGAGGGCAGTGAGACACTGGAACAGGTTTCCCCGTGAGGCTGTGGATGCCCCCTCCCTG
GAAGCACTCCAGGCCAGGCTGGATGGGACGGTGAGCAACCTGGGCTATGGGAGGTGTCCCTGCCTACAGCAG
GGGGGTGGAACGAGGTGGTCTTAA

Figure S1. Sequence before the transcription start site of the chicken ZP2 gene.
